# Highly Selective Fluorescence Sensing and Imaging of ATP Using a Boronic Acid Groups-Bearing Polythiophene Derivate

**DOI:** 10.3390/polym11071139

**Published:** 2019-07-03

**Authors:** Lihua Liu, Linlin Zhao, Dandan Cheng, Xinyi Yao, Yan Lu

**Affiliations:** 1 School of Materials Science & Engineering, Tianjin University of Technology, Tianjin 300384, China; 2 Key Laboratory of Display Materials & Photoelectric Devices, Ministry of Education, Tianjin University of Technology, Tianjin 300384, China; 3 Tianjin Key Laboratory for Photoelectric Materials and Devices, Tianjin University of Technology, Tianjin 300384, China

**Keywords:** ATP detection, fluorescent probe, polythiophene, multisite binding, analyte-induced aggregation

## Abstract

A boronic acid groups-bearing polythiophene derivate (**L**) was designed and synthesized for highly sensitive fluorescence detection of ATP based on a multisite-binding coupled with analyte-induced aggregation strategy. **L** has a polythiophene backbone as fluorophores and two functional side groups, i.e., quaternary ammonium group and boronic acid group, as multibinding sites for ATP. When various structural analogues such as ADP, AMP, and various inorganic phosphates were added into the aqueous solution of **L**, only ATP caused a remarkable fluorescence quenching of about 60-fold accompanied by obvious color changes of solution from yellow to purple. The detection limit is estimated to be 2 nM based on 3*σ*/slope. With the advantage of good water solubility, low toxicity, and highly selective response to ATP, **L** was successfully utilized as a probe to real-time assay activity of adenylate kinase (ADK) and map fluorescent imaging of ATP in living cells.

## 1. Introduction

Nucleotide adenosine triphosphate (ATP) is one of the critical biological anions in all living organisms. It serves as a universal energy source in various cellular events and plays significant roles in many biological processes including biosynthesis, DNA replication, cell signaling, and so on [1,2,3,4,5]. The intracellular ATP levels have been demonstrated to be closely relevant to pathogenesis of many diseases including Parkinson’s disease, ischemia, and hypoglycemia, etc. [5,6,7,8]. Therefore, sensitive and selective detection of ATP is highly desired for biochemical research and clinical diagnosis.

In recent years, fluorescent probes are considered as powerful tools for the visible detection and identification of biological substances owing to the good selectivity, high sensitivity, real-time analysis capability, and high temporal and spatial resolution [9,10,11,12,13,14]. Thanks to the strong light-harvesting and signal-amplification properties of conjugated polymers (CPs), water-soluble CPs-based probes have been demonstrated to be efficient probes for detection of diverse bio-related species such as nucleotides, DNA, proteins, protease, etc. [15,16,17]. Among various CPs, polythiophene (PT) derivatives have received extensive attention because of their sensitive chain conformation transition responsive to external stimuli [7,8,18,19,20,21], providing unique advantages over other CPs on keeping the balance between simplicity (colorimetric mode) and sensitivity (fluorescence mode). In previous reports including ours, several PTs derivatives have been designed for the fluorescent detection of ATP with moderate selectivity and sensitivity [19,22,23,24,25]. These reported PT-based ATP probes contain generally cationic groups on their side chains, for example, quaternary ammonium [18,19,25,26], imidazolium [22], or phosphonium [23], to offer essential water solubility and recognition site for ATP. Thus, in most cases, electrostatic interaction is the primary driving force for the binding of polymer probes with ATP, resulting in these assays being sensitive to ionic strength. This will prevent practical application of these probes in more complex environments like cells and even living body. On the other hand, the selectivity of the reported ATP probe was not satisfying since the electrostatic attraction was nonspecific. To address these challenges, herein, we propose a multisite-binding coupled with analyte-induced aggregation strategy to develop PT-based probes for highly selective ATP detection and imaging.

**Scheme 1:**
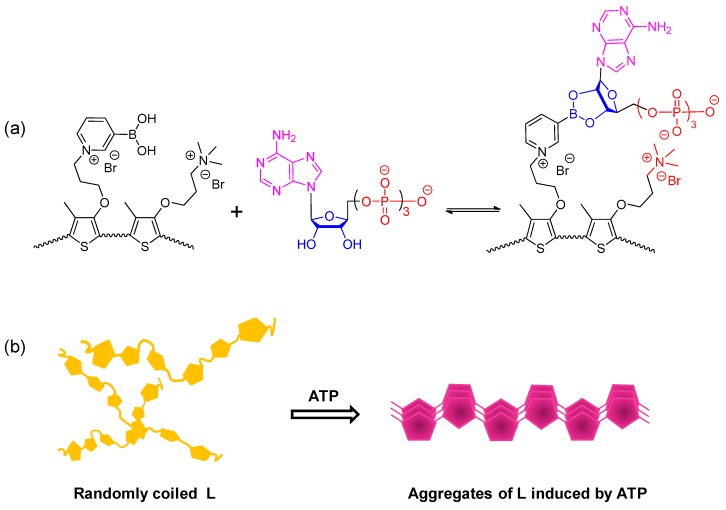
(**a**) The possible binding pattern of **L** with ATP; (**b**) Conformation transition and the formation of aggregates of **L** induced by ATP.

It is well known that boronic acids can bind vicinal diols with high affinities via reversible boronate formation in weak-base aqueous solution, which has been widely used to develop synthetic receptors for saccharides detection [6,26,27,28,29]. In the present contribution, both boronic acid groups and quaternary ammonium groups were introduced to the side chain of polythiophene as binding sites of ribose and phosphate of ATP, respectively, to produce a new ATP probe (**L**) with strong and selective affinity for ATP (Scheme 1a). **L** and ATP come together easily by the electrostatic attraction, at the same time, the neighboring boronic acid group on the side chains of **L** further anchors ATP via formation of pentacyclic borate to restrict the rotation of **L** backbone and thus, promote polymer aggregation through the interchain π–π stacking interaction (Scheme 1b). This process will lead to the significant fluorescence quenching of **L**. Based on the sensing strategy, **L** can distinguish ATP from its analogues ADP and AMP as well as various inorganic phosphates, etc. Taking advantage of high selectivity of **L** to ATP, the activity assay of ATP-related enzyme in buffer solution and the fluorescence imaging of ATP in living cells were successfully achieved.

## 2. Experimental

### 2.1. Materials

Adenosine 5′-riphosphate (ATP), adenosine 5′-diphosphate (ADP), adenosine 5′-nophosphate (AMP), and adenylate kinase (ADK) were provided by Aladdin (Shanghai, China). 3-(4,5-Dimethylthiazol-2-yl)-2,5-diphenyl-tetrazolium bromide (MTT) was purchased from Sigma-Aldrich (St. Louis, MO, USA). 2-Amino-2-hydroxymethyl-1,3-propanediol hydrochloride (*tris*-HCl) was bought from Alfa Aesar (Tianjin, China). The ultrapure water in this study was obtained by a Millipore filtration system (Millipore, Darmstadt, Germany). All other chemicals were used as received without further purification. The polymer concentration was calculated on the basis of the repeat unit.

### 2.2. Instruments

^1^H NMR spectra were performed on a Bruker Avance III-400 NMR spectrometer (400 MHz, Bruker, Fällanden, Switzerland) using tetramethylsilane (TMS) as an internal standard. Gel permeation chromatography (GPC) trace was obtained with a Waters 1515 liquid chromatography system (Waters, MA, USA) equipped with a Waters 2414 refractive index detector (Waters, MA, USA) and Phenogel GPC columns (Waters, MA, USA) using pullulan as the standard and H_2_O as the eluent at a flow rate of 1.0 mL min^−1^ at 35 °C. FTIR spectra were recorded with a ALPHA II FTIR spectrometer (Bruker, Rheinstetten, Germany). Circular dichroism (CD) spectra were collected on a Jasco J-715 WI spectropolarimeter (Jasco, Tokyo, Japan) operating at room temperature. UV-Vis absorption spectra were measured on a Shimadzu UV-2550 spectrometer (Shimadzu, Tokyo, Japan). Fluorescent spectra were recorded with a Hitachi F-4600 fluorescence spectrophotometer (Hitachi, Chiyoda ku, Japan) equipped with a xenon lamp excitation source. Fluorescence quantum yields were achieved by comparison with fluorescien in water as standard. The pH values were determined with a Mettler-Toledo Delta 320 pH meter (Mettler-Toledo, Greifensee, Switzerland).

### 2.3. Synthesis

#### 2.3.1. Synthesis of 3-(3-Bromopropoxy)-4-methylthiophene (**1**)

The monomer **1** was synthesized according the procedure described in the previous work reported by our group [18]. Yield 81%; ^1^H NMR (400 MHz, CDCl_3_) *δ* 6.72 (s, 1H), 6.08 (s, 1H), 3.96 (m, 2H), 3.48 (m, 2H), 2.23 (m, 2H), 1.99 (s, 3H).

#### 2.3.2. Synthesis of 3-(4-Methyl-3′-thienyloxy)propyltrimethylammonium Bromide (**2**)

Trimethylamine (10 mL) was added into a solution of **1** (2.68 g, 11.4 mmol) in THF (100 mL). The mixture was stirred 48 h at room temperature, and then evaporated to dryness. The crude product was washed with THF to give **2** (2.62 g, 78% yield). ^1^H NMR (400 MHz, *d*_6_-DMSO) *δ* 7.01 (s, 1H), 6.48 (s, 1H), 4.15 (m, 2H), 3.51 (m, 2H), 3.14 (s, 9H), 2.30 (m, 2H), 2.06 (s, 3H).

#### 2.3.3. Synthesis of Copolymer (**3**)

Copolymer **3** was synthesized according the procedure as following: anhydrous FeCl_3_ (649 mg, 4 mmol) was added to the freshly dry CHCl_3_ (20 mL), and the mixture was stirred for 30 min at room temperature under N_2_ atmosphere. Then, the solution of **1** (235 mg, 1 mmol) and **2** (294 mg, 1 mmol) dissolved in dry CHCl_3_ (10 mL) was added dropwise to the above as-prepared FeCl_3_ solution. The mixture was stirred for 48 h at 35 °C. The reaction mixture was then concentrated to about 2 mL. The residue was precipitated by addition of MeOH (200 mL). The precipitate was collected by filtration, and the resulting crude product was extracted by Soxhlet extraction with MeOH for 24 h to remove possibly residual FeCl_3_. The residual solid was filtrated and dried under reduced pressure to give copolymer **3** (331.6 mg, 63%). GPC (H_2_O, pullulan standard): *M*_n_: 6.3 kDa, PDI: 1.819.

#### 2.3.4. Synthesis of Copolymer **L**

Copolymer **3** (200 mg) was dissolved in a mixed solvent of DMF (30 mL) and THF (20 mL). 3-Pyridineboronic acid (300 mg, 2.44 mmol) was then added into the above solution. The mixture was stirred at 70 °C under a N_2_ atmosphere for 48 h. The mixed solution was concentrated to about 2 mL, and the residue was then dropwise added into THF (60 mL). The resultant precipitate was collected by filtration, washed with THF (50 mL × 3), and dried under vacuum at room temperature to obtain polymer **L** (211 mg, 86% yield) as a dark-red solid.

### 2.4. Sample Preparation for Spectroscopic Analysis

All spectroscopic experiments were carried out at 25 °C. The stock solution of the probe (**L** = 2.0 × 10^−3^ M) in water was diluted to 50 μM for UV-Vis absorption spectrum and 10 μM for photoluminescence spectrum.

### 2.5. Cell Culture and Cytotoxicity Assays

HeLa cells were cultured in DMEM supplemented with 10% FBS and 1% penicillin–streptomycin at 37 °C in a humidified 5% CO_2_–95% air atmosphere.

The cytotoxicity of **L** was evaluated by typical MTT assay. HeLa cells (1 × 10^4^ cells/well) were seeded onto 96-well plates in 100 μL DMEM and allowed to attach for 24 h. After cell attachment, the medium was removed and the cells were washed with PBS three times, then 100 μL of fresh medium containing **L** with different concentrations ranging from 0 to 50 μM was added and incubated for 24 h. After 24 h incubation, the cell viability was evaluated by MTT assay.

### 2.6. Fluorescence Imaging

HeLa cells (1 × 10^5^ cells/well) were seeded onto petri dish in 2 mL DMEM and allowed to attach for 24 h. After cell attachment, the medium was replaced with 2 mL of fresh medium containing **L** (10 μM, 2 mL) for 2 h, then the medium was removed and the cells were washed with PBS three times. Fluorescence images were recorded with a confocal laser scanning microscope (CLSM) (Olympus FV1000-IX81, Tokyo, Japan). The excitation wavelength used was 405 nm, and all images were analyzed with Olympus FV1000-ASW.

## 3. Results and Discussion

### 3.1. Synthesis of **L**

**Scheme 2:**
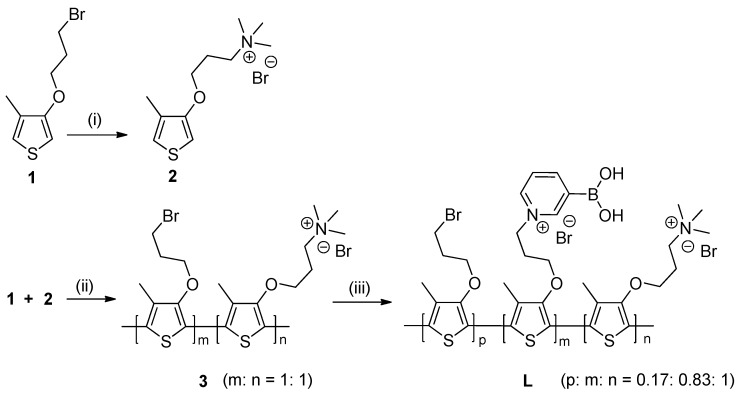
Synthesis of the probe **L**. (**i**) N(CH_3_)_3_, THF, rt, 48 h; (**ii**) FeCl_3_, CHCl_3_, 35 °C, 48 h; (**iii**) 3-Pyridineboronic acid, DMF/THF (3:2 *v*/*v*), N_2_, 70 °C, 48 h.

The synthetic route for the probe **L** is outlined in Scheme 2. The traditional chemical oxidative polymerization with FeCl_3_ in chloroform in the presence of equivalent **1** and **2** gave the random copolymer product **3** with a moderate number-average molecular weight of 6.3 kDa, determined by GPC using pullulan as the standard and H_2_O as the eluent, respectively. Subsequently, 3-pyridineboronic acid groups were linked to the side chain of **3** by nucleophilic substitution reaction to produce the target **L**. The strong absorption peaks at 1,360 and 1,485 cm^−1^ in FTIR of **L** were assigned to the characteristic symmetric and unsymmetric stretching vibration of the B–O bond, respectively (Figure S1, Supplementary Materials), suggesting that the boronic acid groups were successfully introduced into the side chain of **L**. The ratio of the three repeated units in **L** was determined to be 0.17:0.83:1 for Br, boronic acid and quaternary ammonium contained ones, respectively, by inductively coupled plasma (ICP) analysis. In other words, the molar content of boronic acid-containing moieties in the total polymer was about 41.5%.

### 3.2. Screen of pH of Assay System

Since pH values of the aqueous solution influences strongly the formation of ester between boronic acids and vicinal diols [6,26], the pH conditions for ATP sensing using **L** as a probe were first optimized. The pyridinium hydroxyboronate moiety in our probe **L** has a very low pKa value of about 4.0 [30]. Thus, the weakly alkaline conditions with pH values ranging from 7.0–10.0 were carefully assessed. As shown in Figure S2a (Supplementary Materials), the changes on absorption spectra of **L** (50 μM) in water were almost ignored within the total tested pH ranges. Upon addition of 1 equiv. of ATP to **L** aqueous solution (50 μM) with different pH values, the absorption spectra of solutions at pH = 7.4, 8.0, and 9.0 gave the significant responses (Figure S2b, Supplementary Materials). Based on these results, the aqueous solution of physiological pH 7.4 was chosen as assay system for the evaluation of sensing properties of **L**.

### 3.3. Selectivity

**Figure 1:**
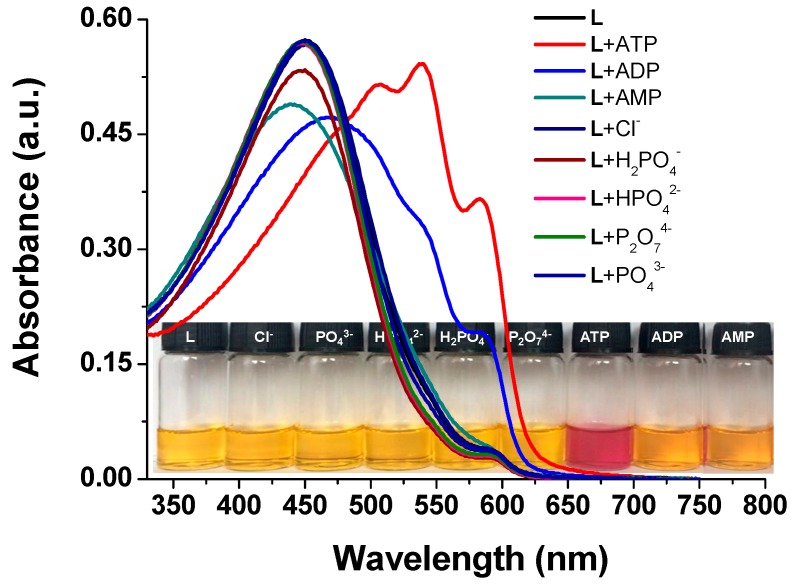
Absorption spectra of **L** (50 µM) upon addition of various bio-related sodium salts of anions including ATP, ADP, AMP, Cl^−^, PO_4_^3−^, HPO_4_^2−^, H_2_PO_4_^−^ and P_2_O_7_^2−^ (50 µM) in water at pH 7.4.

**Figure 2:**
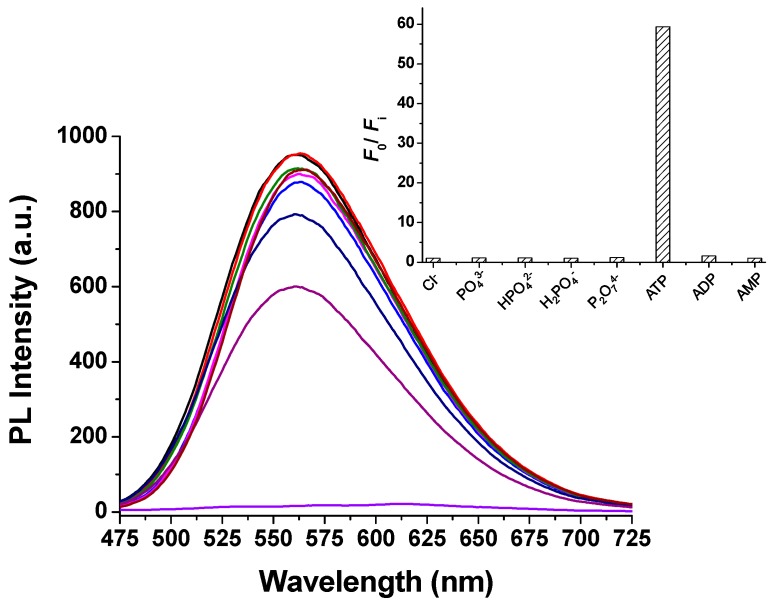
Fluorescent emission changes of **L** (10 µM) upon addition of various bio-related sodium salts of anions including ATP, ADP, AMP, Cl^−^, PO_4_^3−^, HPO_4_^2−^, H_2_PO_4_^−^ and P_2_O_7_^2−^ (30 μM) in water at pH 7.4.

Selectivity of the probes is one of the key parameters for evaluating their performances. We first checked the response of **L** to a wide range of the biologically relevant anions including Cl^−^, PO_4_^3−^, HPO_4_^2−^, H_2_PO_4_^−^, P_2_O_7_^2−^, as well as ATP and its analogues ADP and AMP by absorption and emission spectra. As shown in Figure 1, after addition of 1 equiv. of these anions to aqueous solution of **L**, most of the solutions remained yellow with slight changes of *λ*_max_, except for that containing ADP, which gave orange solutions with the shifts of the absorption peaks from 450 to 468 nm. Compared with those anions, the most remarkable influence was observed upon adding ATP, which gave a purple solution along with the significant red-shifts of *λ*_max_. The significant color change indicated that **L** can serve as a “naked-eye” indicator for ATP in aqueous solution. On the other hand, the addition of 3 equiv. of ATP resulted in almost complete quenching (about 60-fold) in photoluminescence intensity of **L** (Figure 2). However, the addition of other related anions had no obvious effect on the fluorescence emission of **L**, except that adding ADP produced a slight decrease in emission intensity. These results indicated that the probe **L** had high selectivity to ATP over other tested anions in aqueous solution.

### 3.4. UV-Vis Spectral Response of Probe **L** to ATP

**Figure 3:**
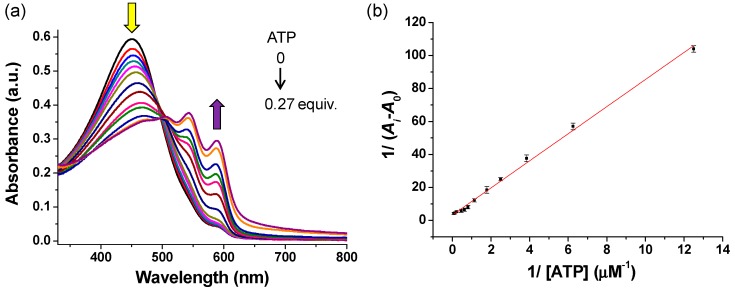
(**a**) UV-Vis absorption spectra of **L** (50 µM) with the addition of ATP with concentrations ranging from 0 to 13.6 µM in *tris*-HCl buffer solution (10 mM, pH = 7.4); (**b**) Absorbance at 540 nm of **L** as a function of ATP concentration. *A*_0_ is the initial absorbance of **L** at 540 nm, and *A*_i_ is the recorded absorbance of **L** in the presence of ATP with different concentrations. Error bars represent the standard deviations of three trials.

The interaction of **L** with ATP in *tris*-HCl buffer solution (10 mM, pH = 7.4) at room temperature was carefully investigated by UV-Vis absorption spectroscopy. As displayed in Figure 3a, the *tris*-HCl buffer solution (10 mM, pH 7.4) of **L** (50 µM) exhibited a maximum absorption band at 450 nm with *ε* = 1.2 × 10^4^ M^−1^ cm^−1^, which is attributed to π–π* transition of polythiophene backbone with a random-coil conformation [19,31,32]. As the concentrations of ATP increased, the *λ*_max_ was gradually red-shifted from 450 to 540 and 588 nm, accompanied with a distinct solution color change from yellow to purple. The significant shift and the appearance of two vibronic bands are characteristic of the aggregation of the polythiophene backbone [19]. Job′s plot (Figure S3, Supplementary Materials) indicated a near 1:1 stoichiometry of complex formed between **L** and ATP. The binding constant of **L** with ATP was estimated to be about 4.02 × 10^5^ M^−1^ with *R*^2^ of 0.995 based on the double-reciprocal plot of 1/(*A* − *A*_0_) versus 1/(ATP), as shown in Figure 3b [33,34,35]. Further, the control homopolymer **PTB,** which just contains boronic acid groups on its side chains, exhibited the binding constant of 2.68 × 10^4^ M^−1^ (Figure S4, Supplementary Materials). In summary, comparing with other PT-based ATP probes which only contained quaternary ammonium groups [18,24] or boronic acid groups on their side chains, the relatively larger binding constant of **L** with ATP implied stronger affinity between the both, probably due to synergistic interactions.

**Figure 4:**
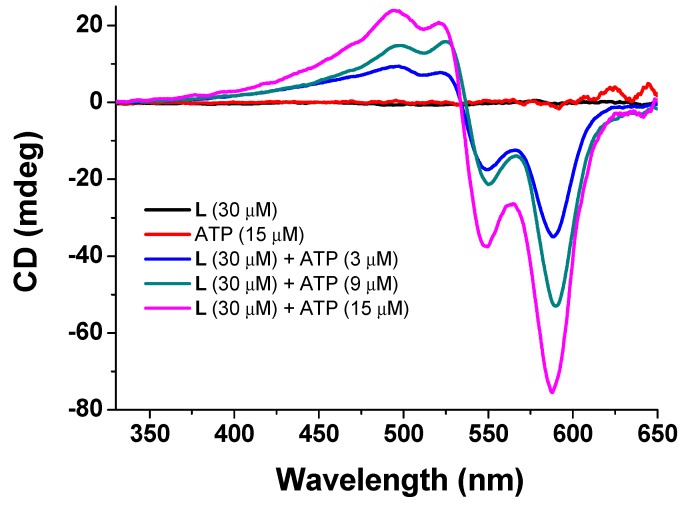
Circular dichroism (CD) spectra of ATP (15 μM) and **L** (30 μM) in the absence and the presence of various amounts of ATP in water (pH 7.4).

Further, we confirmed the formation of **L** supermolecular aggregates induced by ATP by circular dichroism (CD) measurements. As shown in Figure 4, both **L** and ATP are optically inactive, and no CD pattern in the π–π* transition region can be detected. These results indicate that free **L** adopts an achiral random-coiled conformation in water. However, upon binding with ATP, an intense split-type induced CD (ICD) in the π–π* transition region appeared, and as the ATP concentration increased, the ICD intensity increased gradually, suggesting the formation of chiral π-stacked aggregates of **L** in the presence of ATP [36].

### 3.5. Fluorescence Spectral Response of Probe **L** to ATP

**Figure 5:**
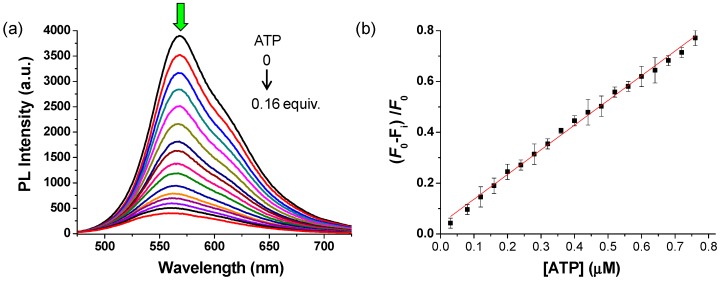
(**a**) Fluorescence spectra of **L** (10 µM) with the addition of various concentrations of ATP in *tris*-HCl buffer solution (10 mM, pH 7.4); (**b**) Plot of (*F*_0_ − *F*_i_)/*F*_0_ at 570 nm vs. concentrations of ATP. *F*_0_ is the initial emission intensity of **L** (10 μM), and *F*_i_ is the recorded emission intensities of **L** in the presence of ATP with different concentrations. Excitation wavelength: 450 nm. Error bars represent the standard deviations of three trials.

Figure 5a displays the changes of fluorescent spectra of polymer **L** (10 µM) in the presence of different amounts of ATP in *tris*-HCl buffer solution (10 mM, pH 7.4). The fluorescence spectra of **L** exhibited a strong fluorescence at 570 nm upon excitation at 450 nm (*φ* = 0.304). The titration of ATP into **L** buffer solution caused that the fluorescent intensities of the emission band centered at 570 nm decreased gradually with the increasing concentrations of ATP. The significant quenching of the emission of **L** could be explained by the formation of π-stacked aggregates of **L** induced by ATP, as demonstrated by UV-Vis and CD studied, quenching light emission via other nonradiative pathways [24,25]. The quenching constant *K*_SV_ was determined according to the well-known Stern-Volmer equation [37]. As seen from the Stern-Volmer plot (Figure 5b), there was a linear relationship between (*F*_0_ − *F*_i_)/*F*_0_, and the concentrations of ATP ranging from 0 to 0.8 μM and *K*_SV_ were estimated to 1.08 × 10^6^ M^−1^ (*R*^2^ = 0.997). The high quenching efficiency indicated the strong affinity between **L** and ATP. In addition, for our system, only 0.16 equiv. of ATP almost completely quenched the fluorescence of **L**, but in case of the PT-based ATP probe bearing only quaternary ammonium groups, about 1 equiv. of ATP was required to produce such a degree of quenching under similar detection conditions [19]. Thus, it is reasonable to believe that boronic acid groups were involved in binding of **L** with ATP, which promoted the conformational transformation and subsequent aggregation of **L**.

From the changes in ATP concentrations-dependent fluorescence intensities (Figure S5, Supplementary Materials), the detection limit was estimated to be 2.07 × 10^−9^ M based on the 3*σ*/slope (where *σ* is the standard deviation of the background and slope is the sensitivity) [24,38]. Such a low detection limit should be very competitive with most of previously reported fluorescence or colorimetric probes for ATP [18,19,20,21,22,23,24,25] (Table S1, Supplementary Materials). The high sensitivity of **L** for ATP should be closely related to the strong synergetic interactions between **L** and ATP.

In order to further confirm that cyclic borate can be formed between 3-pyridineboric acid of **L** and ribose of ATP in weakly basic aqueous solution, ^1^H NMR spectra of 3-pyridineboric acid, ATP, and their mixture at pH 8.0 in D_2_O were collected, as shown in Figure S6. From Figure S6 (Supplementary Materials), it is obviously seen that both the strong peak at *δ* 7.42 in ^1^H NMR of 3-pyridineboronic acid (Figure S6A, Supplementary Materials) assigned as the 5-position H_a_ and the peak at *δ* 6.08 in ^1^H NMR of ATP (Figure S6B, Supplementary Materials) corresponding to H_b_ of ribose in ATP moved towards higher field in ^1^H NMR of 3-pyridineboronic acid/ATP mixture, along with more complex splits (Figure S6C, Supplementary Materials). It could be explained as follows: the neutral form of the boronic acid group serves as an electron-withdrawing group, but after complex with vicinal diols of ribose in ATP, it is transformed into the anionic form, which will act as an electron donor group [28]. These results indicated the formation of covalent bonds between 3-pyridineboronic acid and ATP in a weakly alkaline environment.

### 3.6. Assay for ADK

**Figure 6:**
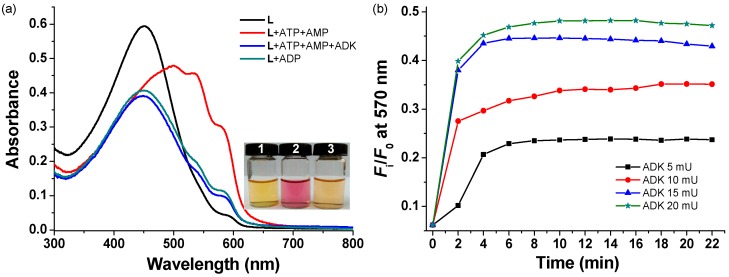
(**a**) Absorption spectra of **L** before and after incubating with ADK in buffer solution. Inset: The photographs taken from **L** (1) and L/ATP/AMP before (2) and after (3) incubating with ADK. (**L**) = 50 μM, (ATP) = (AMP) = 20 μM, (ADP) = 40 μM, (ADK) = 100 mU/mL. Incubating time was 15 min; (**b**) Time-trace plots of the conversion from ATP and AMP to ADP catalyzed by various concentration of ADK (5–20 mU/mL) monitored by the emission ratio *F*_i_/*F*_0_. (**L**) = 10 μM, (ATP) = (AMP) = 2 μM. Excitation wavelength: 450 nm. All measurements were performed in *tris*-HCl buffer solution (10 mM, pH 7.4) containing 0.2 mM Mg^2+^ ions.

Since **L** can selectively recognize ATP from phosphate-containing anions, particularly ADP and AMP, **L** was applied to set up a real-time fluorescence assay for ATP-relevant enzyme activity. For instance, adenylate kinase (ADK) is an important enzyme which is involved in maintaining cellular energy homeostasis. It can catalyze the reversible reaction: ATP + AMP ⇌ 2ADP. As shown in Figure 6a, **L** (50 μM) exhibited an absorption peak at about 450 nm in *tris*-HCl buffer solution (10 mM, pH 7.4) containing 0.2 mM MgCl_2_. When the ATP (20 μM) and AMP (20 μM) were added into the above solution, the absorption band of the solution red-shifted to 535 and 578 nm along with an obvious color change (Inset, Figure 6a). After incubating with ADK ((ADK) = 100 mU/mL) for 15 minutes at 37 °C, the conversion reaction from ATP and AMP to ADP happened, resulting in that the *λ*_max_ returned to 450 nm and the solution color became yellow (Figure 6a). It was interesting to note that the resultant absorption spectra was almost identical with the control spectra of **L** with 40 μM of ADP, indicating the ATP and AMP can be converted to ADP thoroughly under the catalysis of ADK. These results demonstrated preliminarily that **L** can be used as a probe to monitor ADK activity in buffer solution.

Figure 6b shows the dependence of the fluorescence intensity ratio (*F*_i_/*F*_0_) at 570 nm of **L** on the concentration of ADK and incubating time. In this study, the fluorescent spectra were measured at 2 min intervals. From Figure 6b, it is seen that the reaction rate was accelerated with the increase in ADK concentration and after about 10 min, all reaction reached equilibrium. These results indicated that **L** has great potential for facile real-time monitoring of enzyme activity to elucidate their functions in biological systems.

### 3.7. Cell Imaging

Before applying to cell imaging, the biocompatibility of **L** was investigated by MTT assay. As shown in Figure S7 (Supplementary Materials), as the concentration of **L** increased, the average cell viability was greater than 80%, suggesting low cytotoxicity of **L** to living cells in the range of tested concentrations.

The applicability of in situ sensing ATP using **L** as a probe in living cell was further investigated by a confocal laser scanning microscope (CLSM). After incubation of HeLa cells with **L** (10 μM) for 2 h at 37 °C, the strong green fluorescence of **L** was observed in the cells (Figure S8, Supplementary Materials), demonstrating that **L** can be uptaken by HeLa cells with good biocompatibility. The photostability measurement (Figure S8, Supplementary Materials) showed almost negligible changes in the fluorescence intensities of **L** in HeLa cells after continuously irradiating at 405 nm for 100 s.

**Figure 7:**
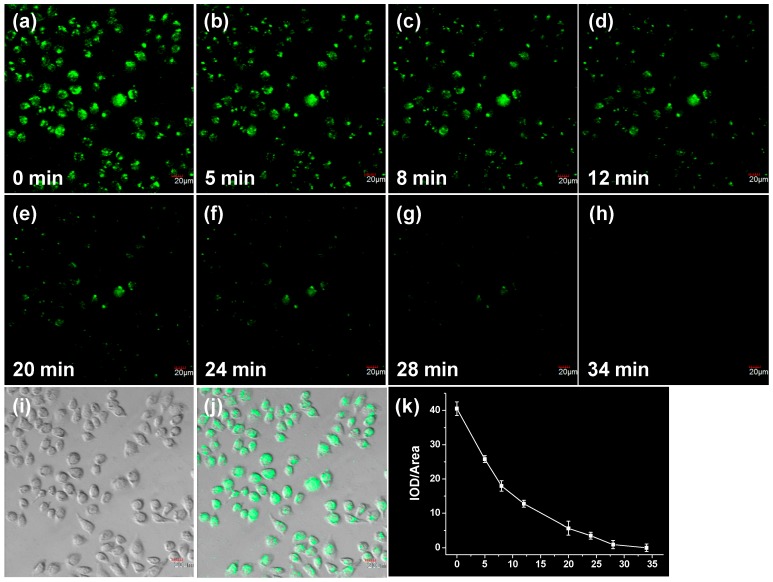
CLSM images of **L**-loaded HeLa cells incubated with ATP (4 μM) for various times from 0 to 34 min (**a**–**h**). (**i**): Brightfield; (**j**): Overlay of (a) and (i); (**k**): Dot representing the integrated optical density (IOD/area) of the probe **L** from the fluorescence images at each time point. Scale bars: 20 μm. *λ*_ex_ = 405 nm, *λ*_em_ = 564–633 nm. Error bars represent the standard deviations of three independent experiments.

The sensing ability of **L** in HeLa cells was subsequently assessed by extracellular addition of ATP (4 μM). Figure 7 shows the representative CLSM images of **L**-loaded HeLa cells that were cocultured with ATP for various times. It was observed that the fluorescence intensity of **L** was decreased gradually with the prolonged incubation time. After about 30 min, the fluorescence of **L** was almost completely quenched. As a control, the fluorescence intensity of **L** itself in HeLa cells did not change significantly within the same test time. These results suggested that **L** had the potential to detect the fluctuation of ATP concentration in living cells.

## 4. Conclusions

A new water-soluble polythiophene-based fluorescent probe **L** with boronic acid groups on its side chains was designed and synthesized for the detection of biologically important ATP. **L** exhibited effective and selective recognition ability for ATP over other structurally similar nucleotides such as ADP and AMP in aqueous solution at physiological pH 7.4. A remarkable fluorescent quenching took place upon addition of ATP into the buffer solution of **L** due to the formation of chiral aggregates induced by ATP via multi-sites binding between **L** and ATP. The change in fluorescent intensities for ATP is much larger than that for ADP and AMP, which is large enough to discriminate ATP from ADP and AMP. Based on the highly selective recognition of **L** for ATP, **L** was successfully utilized as a fluorescent probe for continuous and facile assays of ADK activity. In addition, **L** has been demonstrated to be a useful probe for monitoring the ATP levels in living cells by fluorescence imaging. With the advantages of good water solubility, high selectivity to ATP, and low cytotoxicity, further work to inspect the practical applicability in the real-time detection of enzyme activity in living cells is being carried out in our group.

## Supplementary Materials

The following are available online at https://www.mdpi.com/2073-4360/11/7/1139/s1. Scheme S1. Synthesis of the control polymer **PTB**; Figure S1. FTIR spectra of **L**; Figure S2. Effect of pH on absorption spectra of **L** and **L**/ATP complex; Figure S3. Job’s plot; Figure S4. Determination of the binding constant between **PTB** and ATP; Figure S5. Determination of detection limit of **L**; Figure S6. Study on interaction of 3-pyridineboronic acid with ATP by ^1^H NMR. Figure S7. Cytotoxicity of **L**; Figure S8. Photostability of **L**; Table S1. Comparison of cation or boronic acid-contained polythiophene-based probes for the detection of ATP.
