# Supplementary material for: Highly Selective Fluorescence Sensing and Imaging of ATP Using a Boronic Acid Groups-Bearing Polythiophene Derivate
Source: Polymers (Basel). 2019 Jul 3;11(7):1139. doi: 10.3390/polym11071139 (PMC6680583; doi:10.3390/polym11071139)
Supplement: Supplementary file 1 [file polymers-11-01139-s001.pdf]

Supporting Information

Highly Selective Fluorescence Sensing and Imaging of ATP Using a Boronic Acid Groups-Bearing Polythiophene Derivate

Lihua Liu [1], Linlin Zhao [1,2,*], Dandan Cheng [1], Xinyi Yao [1] and Yan Lu [1,3,*]

1 School of Materials Science & Engineering, Tianjin University of Technology, Tianjin 300384, China
2 Key Laboratory of Display Materials & Photoelectric Devices, Ministry of Education, Tianjin University of Technology, Tianjin 300384, China
3 Tianjin Key Laboratory for Photoelectric Materials and Devices, Tianjin University of Technology, Tianjin 300384, China

*Correspondence: luyan@tjut.edu.cn; luxingzhao@hotmail.com

1. Synthesis of the control polymer PTB

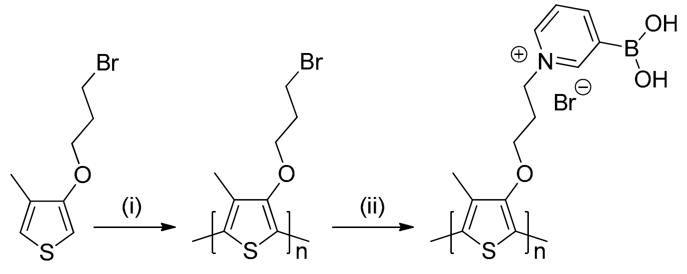

Scheme S1. Synthesis of the control polymer **PTB**. (**i**) $FeCl_3$, $CHCl_3$, 35 °C, 48 h; (**ii**) 3-Pyridineboronic acid, DMF/THF (3:2 *v*/*v*), N_2, 70 °C, 48 h.

*1.1 Synthesis of **PTBr***

Anhydrous $FeCl_3$ (649 mg, 4 mmol) was added to the freshly dry $CHCl_3$ (20 mL), and the mixture was stirred for 30 min at room temperature under N_2 atmosphere. Then the solution of **1** (235 mg, 1 mmol) dissolved in dry $CHCl_3$ (10 mL) was added dropwise the above as-prepared $FeCl_3$ solution. The mixture was stirred for 48 h at 35 °C. The reaction mixture was then concentrated to about 2 mL. The residue was precipitated by addition of MeOH (200 mL). The precipitate was collected by filtration, and the resulting crude product was extracted by Soxhlet extraction with MeOH for 24 h to remove possibly residual $FeCl_3$. The residual solid was filtrated and dried under reduced pressure to give polymer **PTBr** (121 mg, 51%).

1.2 Synthesis of PTB

3-Pyridineboronic acid (209 mg, 1.7 mmol) was added to a solution of polymer PTBr (100 mg) dissolved in a mixture of DMF (15 mL) and THF (10 mL). The flask was degassed and refilled with N_2. The mixture was stirred at 70 °C under a N_2 atmosphere for 48 h. After removing most of the solvent, the residue was dropwise added into THF (60 mL). The precipitate was collected by filtration, washed with THF (50 mL) three times, and dried under vacuum at room temperature to obtain polymer PTB (127 mg, 83.6% yield). GPC (H_2O, Pullulan standard): *M*n 3.7 kDa, PDI 1.415.

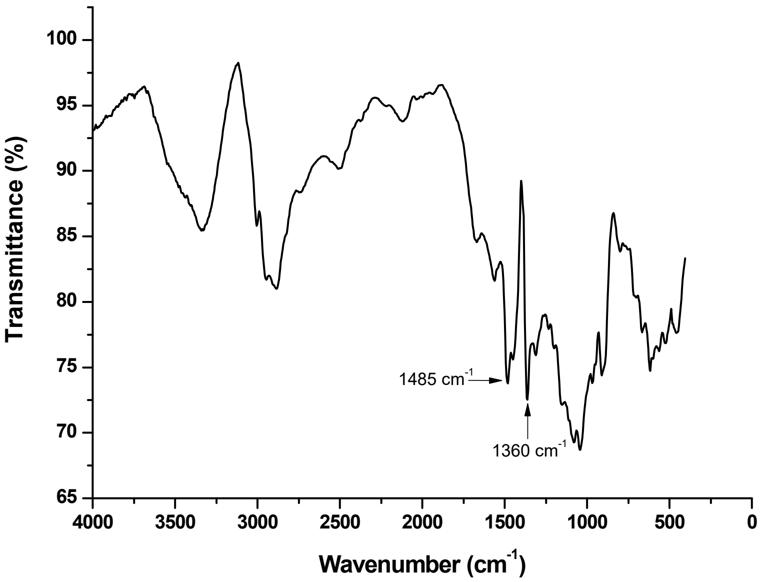

Figure S1. FTIR spectra of **L**.

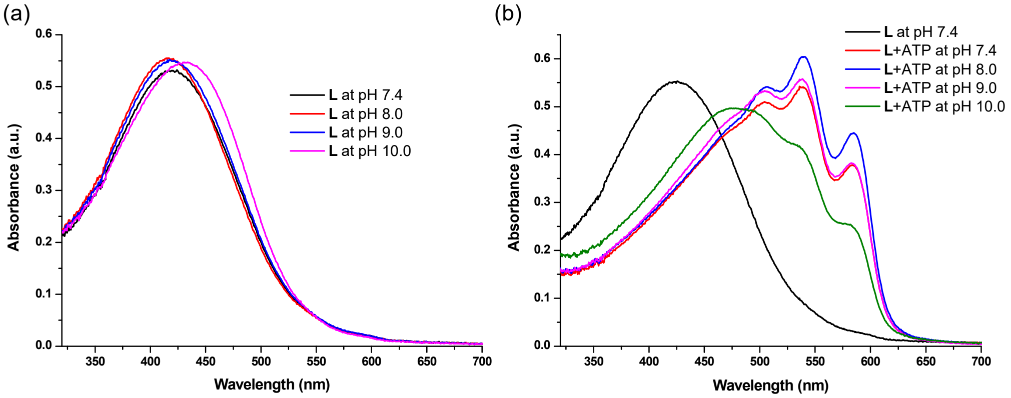

Figure S2. The effect of pH on absorption spectra of **L** and **L**/ATP complex. (**L**) = (ATP) = 50 μM.

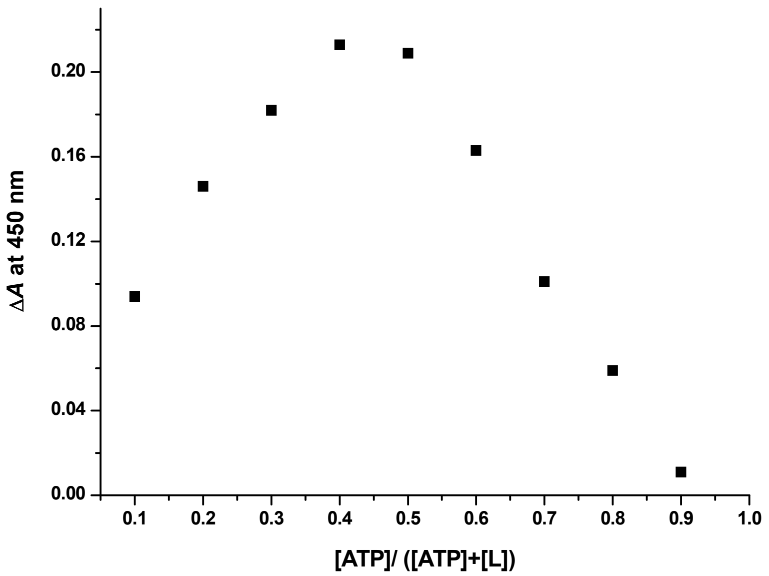

Figure S3. Job's plot.

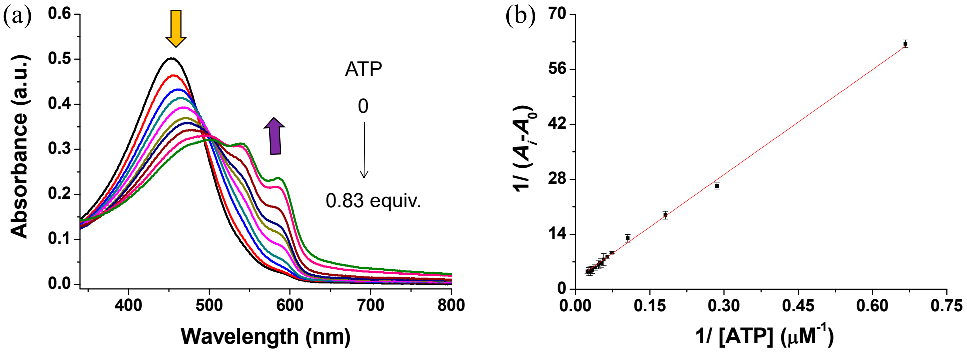

Figure S4. (**a**) UV-Vis absorption spectra of boronic acid homopolymer (50 μM) with the addition of ATP with concentrations ranging from 0 to 41.5 μM in *tris*-HCl buffer solution (10 mM, pH = 7.4); (**b**) Absorbance at 540 nm of boronic acid homopolymer as a function of ATP concentration. A_0 is the initial absorbance of boronic acid homopolymer at 540 nm and A_i is the recorded absorbance of boronic acid homopolymer in the presence of ATP with different concentrations. Error bars represent the standard deviations of three trials.

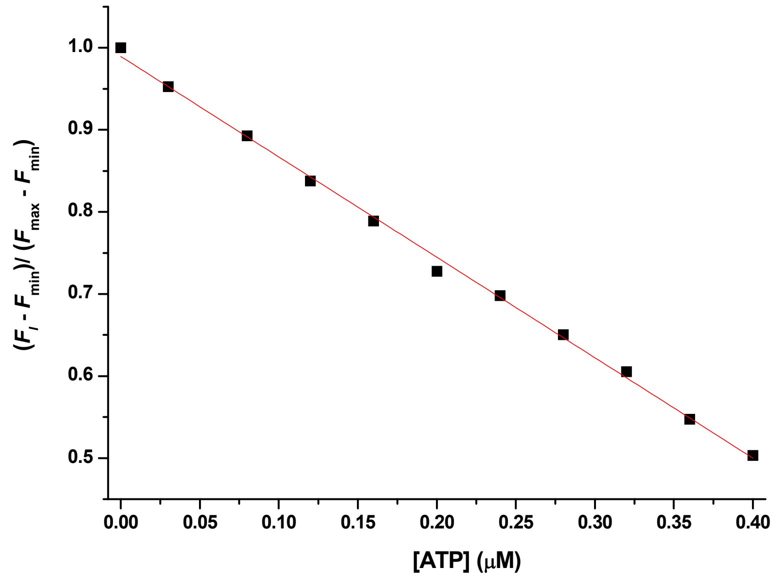

Figure S5. $(F_i - F_{min})/(F_{max} - F_{min})$ as a function of ATP at **L** (10 μM) in *tris*-HCl (10 mM, pH = 7.4) buffer solution.

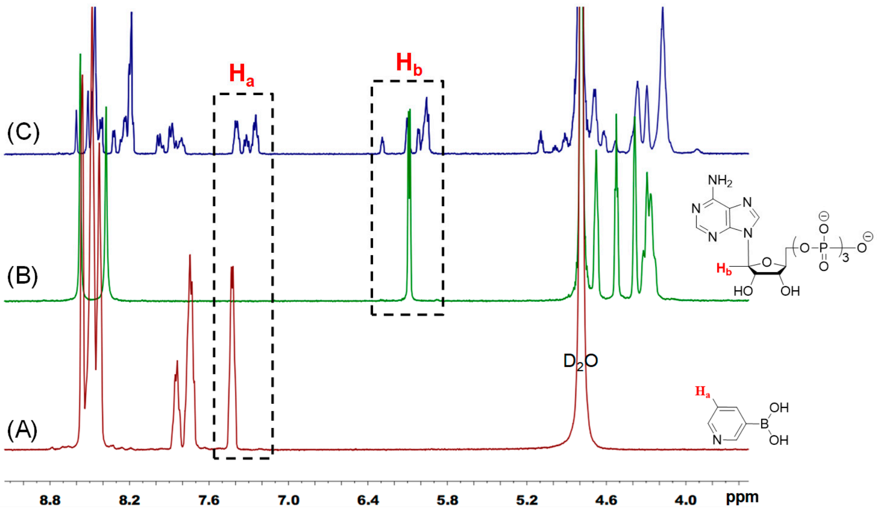

Figure S6. ^1H NMR spectra of 3-pyridineboronic acid (**A**), ATP (**B**) and the mixed solution of ATP (0.01 mmol) and 3-pyridineboronic acid (0.01 mmol) at pH 8.0 (**C**) in D_2O.

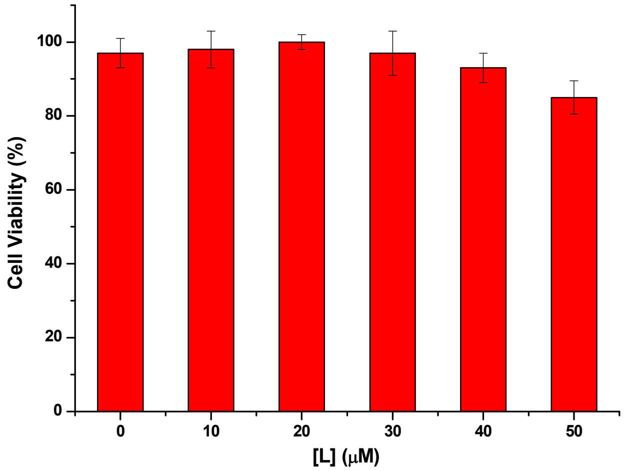

Figure S7. Cell viability (%) of HeLa cells treated with various concentrations of **L** (0–50 μM) estimated by MTT assay. Error bars represent the standard deviations of three independent trials.

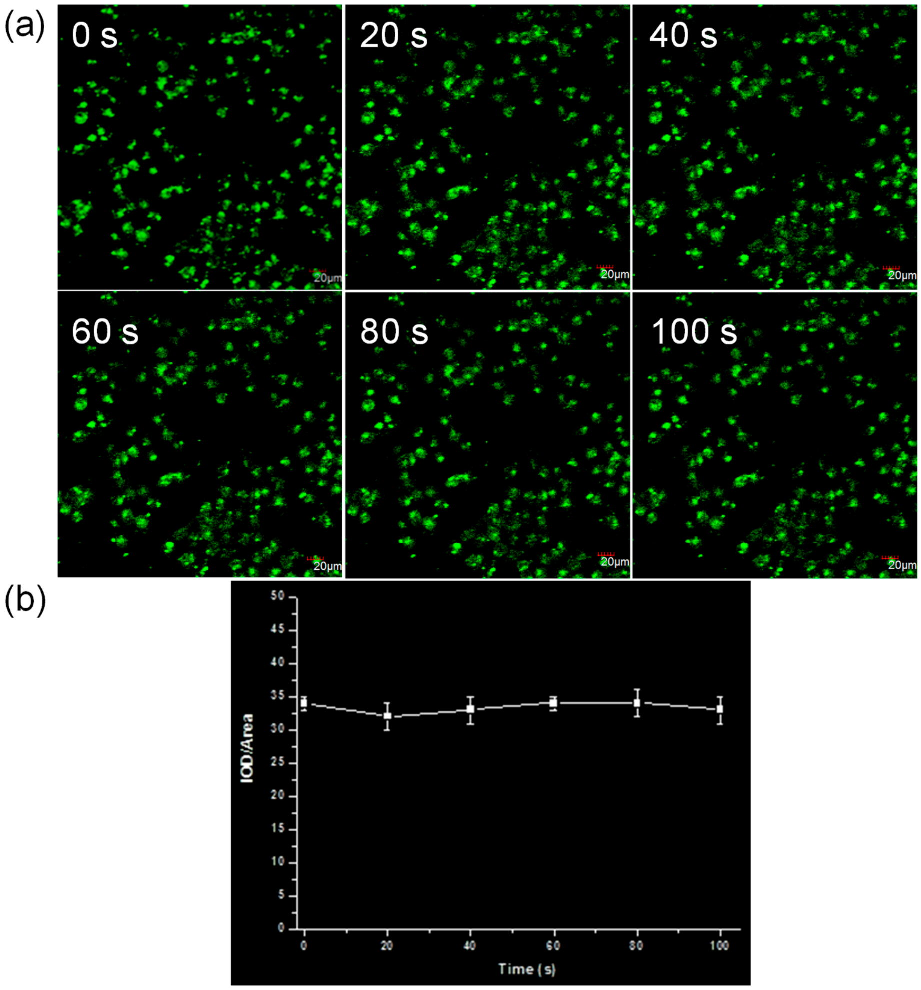

Figure S8. Photostability of **L** (10 μM) co-cultured with HeLa cells for 2 h. λ_{ex} = 405 nm, λ_{em} = 564–633 nm. Error bars represent the standard deviations of three independent experiments.

Table S1. Comparison of cation or boronic acid-contained polythiophene-based probes for the detection of ATP.

Sensor	Detection Limit (M)	Medium of detection	Reference
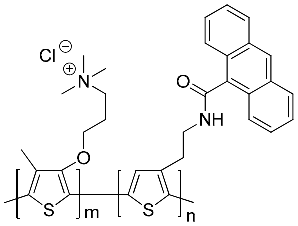	2.3×10^{-9}	*tris*-HCl buffer (pH 7.4)	[18]
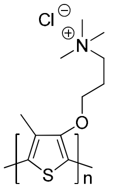	$\sim 10^{-8}$	HEPES buffer (pH 7.4)	[19]
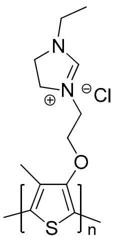	3.6×10^{-11}	HEPES buffer (pH 7.4)	[22]
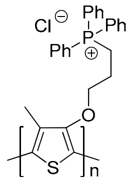	2.7×10^{-8}	HEPES buffer (pH 7.4)	[23]
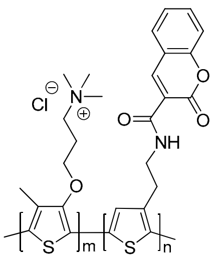	2.9×10^{-8}	*tris*-HCl buffer (pH 7.4)	[24]
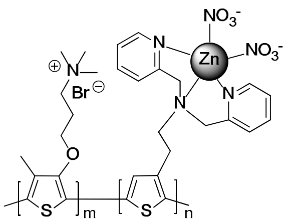	5.2×10^{-9}	*tris*-HCl buffer (pH 7.4)	[25]

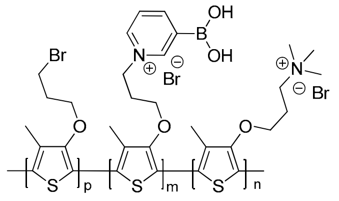 (p: m: n = 0.17: 0.83: 1)	2.07×10^{-9}	*tris*-HCl buffer (pH 7.4)	This paper
